# Association between pelvic obliquity and scoliosis, hip displacement and asymmetric hip abduction in children with cerebral palsy: a cross-sectional registry study

**DOI:** 10.1186/s12891-020-03484-y

**Published:** 2020-07-14

**Authors:** Gunnar Hägglund

**Affiliations:** Department of Clinical Sciences, Orthopedics, Lund University, Skane University Hospital, 221 85 Lund, Sweden

**Keywords:** Cerebral palsy, Pelvic obliquity, Scoliosis, Hip displacement, Hip abduction

## Abstract

**Background:**

Pelvic obliquity (PO) is common in individuals with cerebral palsy (CP). The prevalence of PO in a population of children with CP and its associations with scoliosis, hip displacement and asymmetric range of hip abduction were analysed.

**Methods:**

Over a 5-year period all pelvic radiographs from the Swedish surveillance programme for CP and the recorded data for scoliosis and hip abduction in children from southern Sweden at Gross Motor Function Classification System (GMFCS) levels II–V were analysed. PO and hip displacement calculated as migration percentage (MP) were measured on supine pelvic radiographs and compared with presence of scoliosis and side difference in hip abduction. Statistical analyses comprised chi-squared and binominal testing.

**Results:**

In total, 337 children were analysed, of whom 79 had a PO of ≥5°. The proportion of children with PO increased from 16% in GMFCS level II to 34% in level V. Scoliosis combined with PO was reported in 38 children, of whom 34 (89%, 95% confidence interval [CI] 80–99%) had the convexity opposite to the high side of the PO. Asymmetric abduction with PO was reported in 45 children, of whom 40 (89%, CI 79–99%) had reduced abduction on the high side of the PO. Asymmetric MP of ≥5% with PO was seen in 62 children, of whom 50 had higher MP on the high side of the PO (81%, CI 71–90%). Children in GMFCS levels II–IV more often had isolated infra-pelvic association with PO (47% versus 19% in GMFCS V, *P* = 0.025), while combined supra- and infrapelvic association was more common in GMFCS level V (65% versus 30% in GMFCS II–IV, *P* = 0.004). Isolated infrapelvic or no association was seen in 74% of children ≤10 years of age while 76% of children ≥11 years old had suprapelvic or combined supra- and infrapelvic association with PO (*P* < 0.001).

**Conclusions:**

There was a strong association between the high side of the PO and the side of scoliosis, highest MP, and lowest range of abduction when PO was measured in supine position. PO was more often associated with infrapelvic factors. PO was seen in young children indicating the need for early awareness of this complication.

## Background

Pelvic obliquity (PO), a common deformity in individuals with cerebral palsy (CP), is defined as an asymmetry of the pelvis in the frontal plane and can be measured clinically or radiographically in standing, sitting or lying positions. PO can result in pain, pressure ulcers and difficulties in maintaining sitting and standing postures with reduced functional abilities [1, 2]. The causes of PO can be either supra- or infrapelvic or both. Scoliosis in CP is often thoracolumbar and sometimes the pelvis is involved in the curve, manifesting as a PO with the convexity most often, but not always, opposite to the high side of the PO [2]. Asymmetric ranges of hip abduction or hip displacement are reported as infra-pelvic causes of PO. However, the evidence of any relationship between these factors has been contradictory and inconclusive [2–4].

The Swedish CP Follow-Up Program (CPUP) is a surveillance programme, which monitors more than 95% of the total population of children with CP in Sweden [5, 6]. CPUP includes annual standardised clinical examinations of the spine, range-of-motion measurement of the hips and radiographic follow-up of the hips with pelvic anteroposterior pelvic views taken in supine position.

The primary aim of this study was to analyse the prevalence of PO in a population of children in a hip surveillance programme at different ages and and gross motor function levels. The second aim was to analyse the associations between PO, scoliosis, hip displacement, and range of hip abduction.

## Methods

In CPUP, the diagnosis of CP is verified by a neuropaediatrician after the child turns 4 years old. Gross motor function is classified by the child’s physiotherapist according to the expanded and revised Gross Motor Function Classification System (GMFCS-E&R) [7]. GMFCS-E&R is an age-related five-level system in which children at level I are the least affected and those at level V are the most affected. CPUP includes standardized repeated anteroposterior pelvic radiographic examinations. Children in GMFCS levels III–V undergo annual radiographic examination and those in level II are examined at 2 and 6 years of age. Children in level I are not examined radiographically provided that the physiotherapist’s reports show a normal pain-free range of hip motion. After 8 years of age, the children are followed individually based on the results of the previous radiographic and clinical reports, usually every 12–24 months until skeletal maturity.

**Fig. 1:**
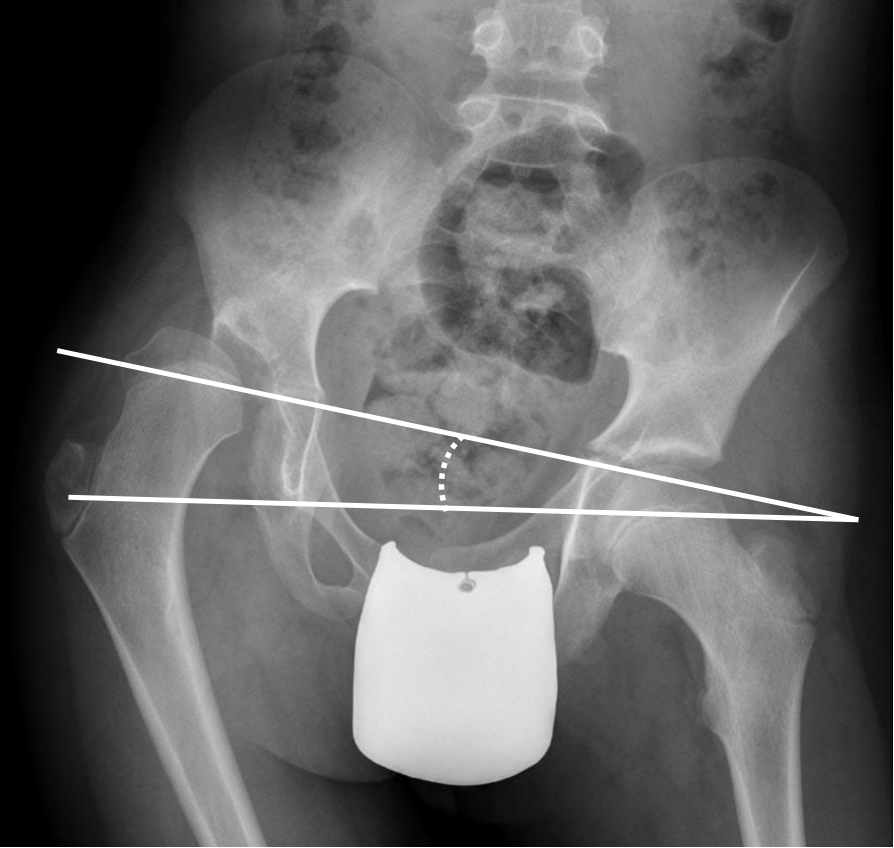
Measurement of pelvic obliquity (PO)

In this cross-sectional study, all children in GMFCS levels II-V up to 18 years of age in southern Sweden (Skåne and Blekinge, comprising 1.4 million inhabitants) who had a radiographic hip examination performed during a 5-year period from 1 July 2014 to 31 June 2019 were analysed. Children in GMFCS level I were not included due to limited number of participants. For children who underwent repeated radiographic examinations during the study period, the first examination was used. Children treated by varus osteotomy of the proximal femur and/or scoliosis surgery before the first examination were excluded. The PO was measured with a line joining the maximum prominence of the ischial tuberosities or through the inferior aspect of both triradiate cartilages (Hilgenreiner’s line) and the horizontal reference frame of the radiograph (Fig. 1). Hip displacement was measured using Reimer’s migration percentage (MP) [8].

Information about scoliosis and range of hip abduction was collected from the physiotherapist’s report performed the same year as the radiographic examination. The spine was assessed in a sitting position, upright and with forward bending. Any spinal deviation was graded as mild, moderate or severe scoliosis according to guidelines outlined in the CPUP manual [9]. This standardized clinical spinal assessment has shown high inter-rater reliability, sensitivity, specificity, and criterion-related validity compared with radiographic Cobb angle measurement [10]. Range of hip abduction was measured in the supine position according to the CPUP manual. The stationary arm of the goniometer was placed parallel to a line joining the two superior iliac spines and the movable arm parallel to the longitudinal axis of the femur.

The proportion of children with of PO was calculated at different ages and GMFCS levels. PO was also calculated related to the presence of scoliosis (= supra-pelvic association) and the association between the high side of the PO and the convexity of the scoliosis was analysed. In children with S-shaped scoliosis, the direction of the lower curve was recorded. PO was finally calculated related to the difference in hip abduction and MP between the hips on the high and low sides of the PO (= infra-pelvic association). The children with PO were analysed by two-sided binomial tests [11] with the null hypothesis 0.50 concerning scoliosis, difference in range of hip abduction, or MP including only those with a difference in range of motion (ROM) of ≥ 5° or a difference in MP of ≥ 5%. The proportions were described with 95% confidence intervals. The chi-squared test was used to analyse differences between groups.

The study was approved by the Medical Research Ethics Committee at Lund University (LU-443-99).

## Results

During the 5-year study period, 385 individuals (209 boys, 176 girls) were examined radiographically. Of these, 38 children (18 boys, 20 girls) had been treated by varus osteotomy, 9 (4 boys, 5 girls) for scoliosis and 1 girl had undergone both varus osteotomy and spine surgery before the first examination. These 48 children were excluded from the study, leaving 337 individuals (187 boys, 150 girls) remaining in the analysis. Their age at examination and GMFCS distribution are presented in Table 1.

**Table 1:** Distribution of age and Gross Motor Function Classification System (GMFCS) level

Age (years)	GMFCS level				Total
	II	III	IV	V	
< 3	4	5	7	10	26
3–4	31	13	11	13	68
5–6	13	13	15	12	53
7–8	25	5	13	13	56
9–10	6	7	14	5	32
11–12	4	12	12	9	37
13–14	3	7	12	8	30
15–16	0	8	10	3	21
17–18	1	5	5	3	14
Total	87	75	99	76	337

**Fig. 2:**
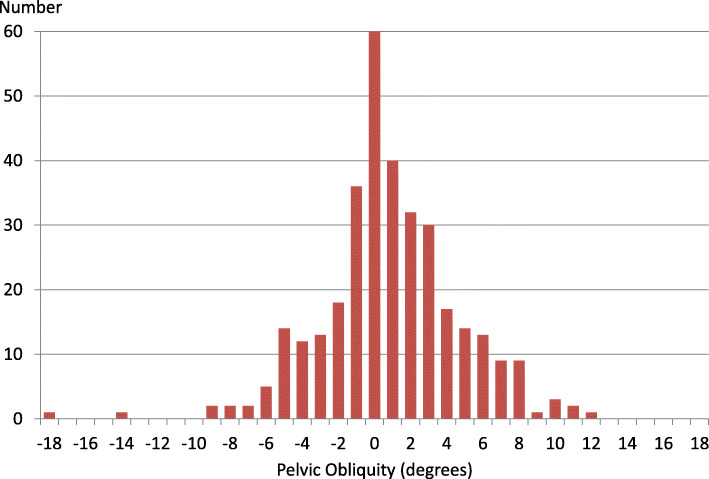
Number of pelvic radiographs related to degree of pelvic obliquity. Negative value = left side of pelvis high, positive value = right side of pelvis high

In the study cohort of 337 children, the PO ranged between 0° and 18°. The left side was higher in 106 cases, the right in 171, and 60 radiographs showed no PO (Fig. 2). The PO was ≥5° on 79 radiographs (left side high in 27, right in 52).

**Fig. 3:**
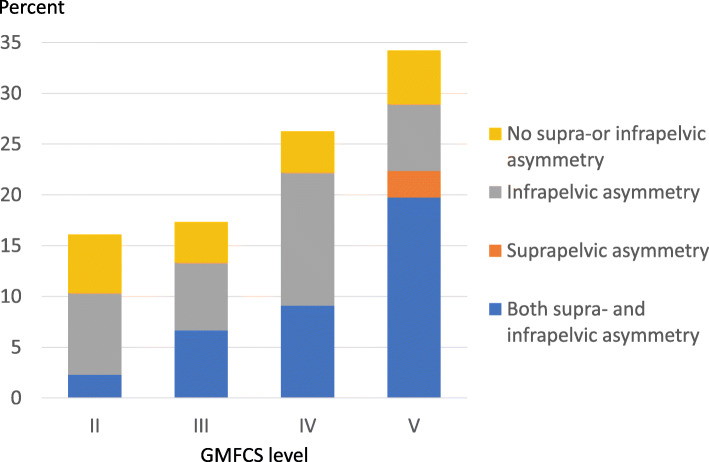
Proportion of children with PO ≥ 5% at different GMFCS levels. Proportion with supra-pelvic (scoliosis) and infra-pelvic (hip displacement or hip abduction)-associated asymmetry indicated

**Fig. 4:**
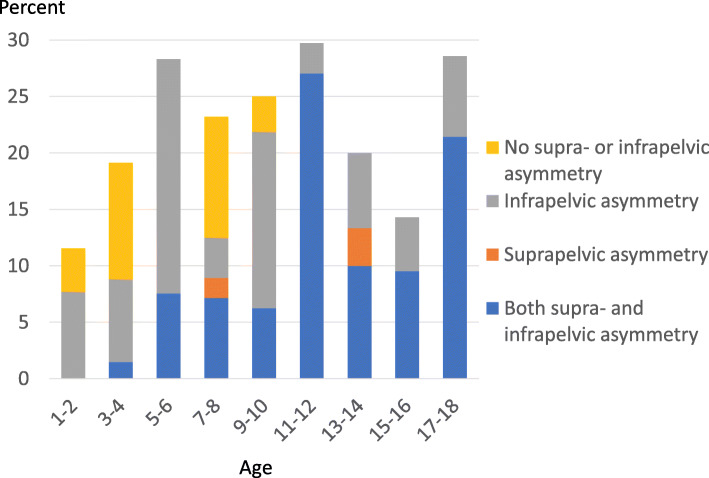
Proportion of children with PO ≥ 5% in different age groups. Proportion with supra-pelvic (scoliosis) and infra-pelvic (hip displacement or hip abduction)-associated asymmetry indicated

The number of children with PO increased with each GMFCS level. The proportion of children with PO ≥ 5° was 16% in level II, 17% in level III, 26% in level IV and 34% in level V (Fig. 3). The number of children with PO ≥ 5° varied with age (Fig. 4).

Scoliosis was reported in 95 children, graded as mild (*n* = 45), moderate (*n* = 30) or severe (*n* = 20). The number of children with PO ≥ 5° and mild scoliosis was 13 (29%), moderate scoliosis 13 (43%) and severe scoliosis 12 (60%). The curve convexity was at the opposite side to the high level of the PO in 9 of the 13 hips with mild scoliosis and in all 25 hips with moderate and severe scoliosis. The proportion of children with curve convexity opposite the high side of PO was 34/38 = 89% (95% CI 80–99%).

The range of hip abduction among the 79 children with PO ≥ 5° was ≥5° lower on the high side of the PO in 40 children, ± 4° in 34 and ≥ 5° higher in 5 children. The difference in ROM for all the 5 children with higher range of abduction on the high side of PO was 5°. The proportion of children with ≥5° lower range of hip abduction on the high side of PO was 40/45 = 89% (95% CI 79–99%).

The MP was ≥5% lower on the high side of the PO in 50 children, ± 4% in 17 children and ≥ 5% higher on the low side of PO in 12 children. The proportion of children with ≥5% lower MP on the high side of PO was 50/62 = 81% (95% CI 71–90%).

**Fig. 5:**
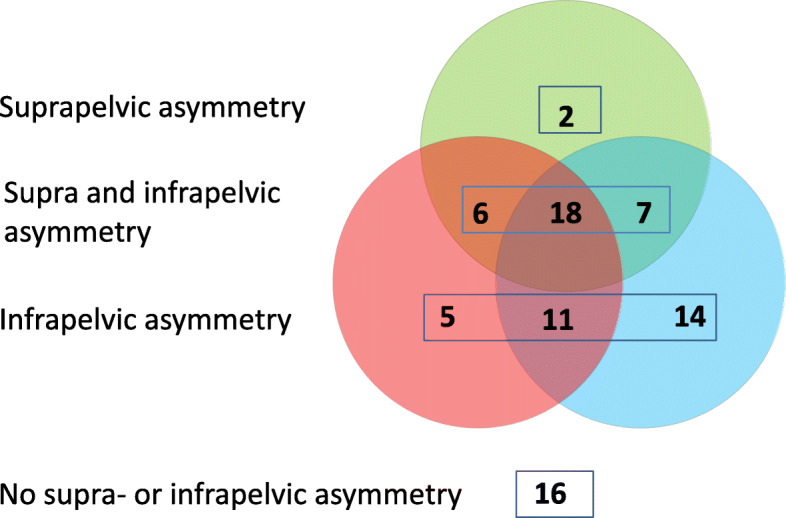
Venn diagram showing the number of children with PO ≥ 5° and scoliosis with convexity opposite to the high side of PO (green), reduced hip abduction (red) and/or higher MP (blue) on the high side of PO. Sixteen children had PO without associated asymmetry

Isolated combination of supra-pelvic asymmetry and PO, i.e., scoliosis with the curve deviation to the low side of PO, without higher MP or lower range of abduction on the high side of PO, was seen in 2 children. Isolated combination of infra-pelvic asymmetry with PO, i.e., higher MP or lower range of abduction on the high side without scoliosis, was seen in 30 children. Both supra- and infra-pelvic asymmetry and PO was seen in 31 children and in 16 children neither supra- nor infra-pelvic asymmetry was seen (Fig. 5).

Children in GMFCS levels II–IV more often had isolated infra-pelvic asymmetry in combination with the PO (25/53 = 47% versus 5/26 = 19% in GMFCS V, *P* = 0.025), while combined supra- and infra-pelvic asymmetry was more common in GMFCS level V (17/26 = 65% versus 16/53 = 30% in GMFCS II–IV, *P* = 0.004) (Fig. 3). Of children ≤10 years of age, 40/54 (74%) had infra-pelvic or neither infra- nor supra-pelvic asymmetry in combination with PO compared with 6/25 (24%) children aged ≥11 years. The remaining 14/54 (26%) children aged ≤10 years and 19/25 aged ≥11 years (76%) had supra-pelvic or both supra- and infra-pelvic asymmetry and PO (*P* < 0.001) (Fig. 4).

## Discussion

The main finding of this study was the strong association between the high side of PO and the convexity of scoliosis, the hip with the highest hip displacement (MP) and the most reduced range of hip abduction.

Porter et al. [2] analysed 747 individuals with CP, all in GMFCS level V. They measured PO in a sitting position and found a strong association between the convex side of scoliosis and low side of PO, but no significant association between PO and the side of hip displacement. Lonstein and Beck [3] reported similar results among 304 individuals with CP classified as dependent sitters, reporting no correlation between the high side of PO, measured in a sitting position, and the side of hip displacement. Letts et al. [4] in a longitudinal study of 22 children with hip displacement, PO and scoliosis found that all hip displacement occurred on the high side of the PO, measured in both sitting and supine positions. In the present study all measurements of PO were made with the child in a supine position, which probably explains the differences compared with earlier studies that measured the PO in a sitting or varied sitting or supine positions. PO caused by rigid scoliosis is seen both in sitting and lying positions. PO caused by asymmetric hip abduction is visible only in lying or standing positions with extended hips. In a sitting position, the legs deviate aside, or the pelvis rotates backwards on the adducted side, but it does not produce a PO.

The higher frequency of PO seen in the children with lower levels of motor function is consistent with the known higher frequency of scoliosis, hip dislocation, windswept position and reduced range of hip motion in children at higher GMFCS levels [12–14].

There was no clear age trend for PO. The children excluded because of surgery were usually > 6 years of age, which might bias the results. PO was also common among children < 5 years old, supporting the importance of early follow-up of these deformities [15]. There were 60 children who had PO without scoliosis, asymmetric abduction or MP. Most of them were young (Fig. 4). These children may have had a side difference in muscle tone that caused PO but so far no fixed deformities.

PO was more often seen in combination with hip displacement or asymmetric range of abduction than in combination with scoliosis. There were more children excluded due to femoral varus osteotomy (*n* = 39) than scoliosis (*n* = 10) indicating that the dominance of infra-pelvic association would have been even greater if these children had been included. The children are followed in a hip surveillance programme in which several have been treated with preventive adductor–psoas tenotomy, which also may have reduced the number of POs with infra-pelvic aetiology. The fact that more young children have infra-pelvic association with PO also indicates that PO is more often initiated by asymmetric hip abduction or asymmetric hip displacement than by scoliosis. This is supported by the longitudinal study reported by Letts et al. [4], which showed that in most patients with hip dislocation, PO and scoliosis, the hip dislocation came first, followed by PO and then by scoliosis.

PO can cause major problems, and is often difficult to treat. PO uncovers the femoral head on the high side of the obliquity which might accelerate a hip displacement. PO in combination with unbalanced scoliosis causes an unbalanced sitting position with higher pressure on the low side of the obliquity with risk of pain and decubitus ulceration. It is therefore important that PO is prevented as far as possible by symmetrical and varied positions of lying and sitting from an early age [15], and that the presence of PO is considered in decisions about spine or hip surgery. The presence of PO should be recorded in hip surveillance programmes. One way to account for the PO is to measure the pelvic adjusted MP (PAMP) [16].

There are several limitations to this study. The cross-sectional design means that we cannot prove any causal relationship between PO and scoliosis and infra-pelvic asymmetries. Scoliosis was assessed by clinical examination. However, this standardized assessment has shown high sensitivity, inter-rater reliability and validity compared with radiographic Cobb angle measurement [10]. The study is based on the total population of children with CP in the area, which is a major strength. However, those patients (48/385) treated with spine and hip surgery were excluded, several of whom probably had PO.

## Conclusion

In summary, PO measured in the supine position had a strong association with scoliosis, hip displacement and asymmetric range of hip abduction, in which the high side of the PO was usually opposite to the convex side of the scoliosis, and on the same side as the largest hip displacement and the lowest range of abduction. PO was more often associated with hip displacement and/or asymmetric range of hip abduction than with scoliosis, and this association was more commonly seen in younger children, indicating that PO most often has an infra-pelvic cause. The presence of PO should be recorded in hip surveillance programmes and as far as possible prevented.

## Data Availability

Data used in this study are stored at the National Quality Register CPUP http://rcsyd.se/anslutna-register/cpup. Data are not publicly available and permission to extract data can be obtained from the register owner.

## Abbreviations

CI: Confidence Interval
CP: Cerebral Palsy
CPUP: Cerebral Palsy Follow-Up Programme
MP: Migration Percentage
PO: Pelvic Obliquity
GMFCS: Gross Motor Function Classification System
